# Phytochemical Composition and Antimicrobial Activity of Essential Oil from the Leaves of *Artemisia vulgaris* L.

**DOI:** 10.3390/molecules28052279

**Published:** 2023-02-28

**Authors:** Nameirakpam Bunindro Singh, Moirangthem Lakshmipriyari Devi, Thokchom Biona, Nanaocha Sharma, Sudripta Das, Jharna Chakravorty, Pulok Kumar Mukherjee, Yallappa Rajashekar

**Affiliations:** 1 Insect Resources Laboratory, Institute of Bioresources and Sustainable Development, Department of Biotechnology, Government of India, Imphal 795001, India; 2 Department of Zoology, Rajiv Gandhi University, Itanagar 781014, India; 3 Plant Molecular Genetics and Genomics Laboratory, Institute of Bioresources and Sustainable Development, Department of Biotechnology, Government of India, Imphal 795001, India

**Keywords:** *Artemisia vulgaris*, essential oil, gas chromatography/mass spectrometry, solid-phase microextraction, eucalyptol, antimicrobial

## Abstract

*Artemisia vulgaris* is an enormously useful aromatic plant known for its insecticidal, antifungal, parasiticidal, and medicinal values. The main aim of this study is to investigate phytochemical contents and the potential antimicrobial activities of *Artemisia vulgaris* essential oil (AVEO) from the fresh leaves of *A. vulgaris* grown in Manipur. The AVEO isolated by hydro-distillation from *A. vulgaris* were analyzed by gas chromatography/mass spectrometry and solid-phase microextraction-GC/MS to describe their volatile chemical profile. There were 47 components identified in the AVEO by GC/MS, amounting to 97.66% of the total composition, while 97.35% were identified by SPME-GC/MS. The prominent compounds present in AVEO analyzed by direct injection and SPME methods are found to be eucalyptol (29.91% and 43.70%), sabinene (8.44% and 8.86%), endo-Borneol (8.24% and 4.76%), 2,7-Dimethyl-2,6-octadien-4-ol (6.76% and 4.24%), and 10-epi-γ-Eudesmol (6.50% and 3.09%). The consolidated component in the leaf volatiles comes to the terms of monoterpenes. The AVEO exhibits antimicrobial activities against fungal pathogens such as *Sclerotium oryzae* (ITCC 4107) and *Fusarium oxysporum* (MTCC 9913) and bacterial cultures such as *Bacillus cereus* (ATCC 13061) and *Staphylococcus aureus* (ATCC 25923). The percent inhibition of AVEO against the *S. oryzae* and *F. oxysporum* was found up to 50.3% and 33.13%, respectively. The MIC and MBC of the essential oil tested for *B. cereus* and *S. aureus* were found to be (0.3%, 0.63%) and (0.63%, 2.5%), respectively. Finally, the results revealed that the AVEO characterized by the hydro-distillation and SPME extraction yielded the same chemical profile and showed potent antimicrobial activities. Further research into *A. vulgaris’s* antibacterial properties can be performed in order to use it as a source for natural antimicrobial medications.

## 1. Introduction

*Artemisia vulgaris* L. is a rhizomatous perennial weed that is heavily invasive in the landscape, agronomic environment, waste sites, and along roadsides [1]. One of the significant therapeutic plant species in the genus *Artemisia* is *A. vulgaris,* which is typically recognized for its volatile oil. Recent studies have demonstrated that this species has antioxidant, hypolipidemic, antispasmodic, analgesic, estrogenic, cytotoxic, antibacterial, antifungal, hypotensive, and broncholytic properties [2,3,4]. Majority of these activities are related to the existence of several groups of secondary metabolites, including flavonoids, sesquiterpene lactones, coumarins, acetylenes, phenolic acids, organic acids, and mono- and sesqui-terpenes [5]. *A. vulgaris* is a folk medicinal plant, which is cosmopolitan in the *Asteraceae* family [6]. *A. vulgaris* is consumed as food and flavoring agent, culinary herbs for poultry, acupuncture therapy, analgesic agent, emmenagogue, and for numerous medicinal purposes, such as anti-epileptic, carminative, anti-inflammatory, antispasmodic, anthelminthic, etc. [7,8]. In many cases, *Artemisia* species predominately produce monoterpenes, including *A. vulgaris* [9]. Traditionally, *A. vulgaris* is known for its insecticidal, parasiticidal, and antimicrobial properties in India.

The northeast region of India belongs to a humid subtropical climate caused by hot, severe monsoons, humid summers, and mild winters, which supports diverse flora and fauna and several other crop species [10]. The wild-growing *Artemisia* species in northeast India has a few ethnobotanical studies, including the chemical profiling of the essential oils, and climate change and its impact on medicinal plants, thereby resulting in noticeable changes in the lifecycles, phenological shifts, distribution of the plant species, and the secondary metabolites [11]. Therefore, it is important to explore active constituents of the essential oil or extracts from medicinal plants, subject to change, and that could be brought to use as medicines in the future.

Essential oils have a huge potential in the field of biomedicine because of their effectiveness in treating a wide range of bacterial, fungal, and viral disorders. Since they contain diverse types of aldehydes, phenolics, terpenes, and other antibacterial components, essential oils are effective against a variety of ailments [12]. Earlier studies have shown that AVEO possesses antimicrobial properties and can be used for various medicinal purposes. In addition, the essential oil of *Artemisia* species was also found to possess antifungal activities against certain *Fusarium* species, such as *F. moniliforme, F. solani,* and *F. sporotrichioides* [13,14].

The current study aims to investigate and develop a technique for profiling the volatile chemical composition using two different methods, direct injection of the essential oil and solid-phase microextraction (SPME), as well as to study the antimicrobial activity of the essential oil against two plant pathogens, *F. oxysporum* and *S. oryzae*, as well as against pathogenic bacteria such as *S. aureus*, *B. cereus, E. coli,* and *P. aeruginosa.*

## 2. Results

The essential oils were isolated from the fresh leaves of *A. vulgaris* by hydro-distillation. The oil content in the leaves of *A. vulgaris* was 0.75% (*w/v*), with eucalyptol as the most prominent compound. The same essential oils were investigated for their chemical compositions using both the oil sample injection as well as by SPME method. The compositions of the AVEO were determined by gas chromatography–mass spectrometry. The AVEO detected in the oil injection and headspace extractions are shown in Table 1.

**Figure 1:**
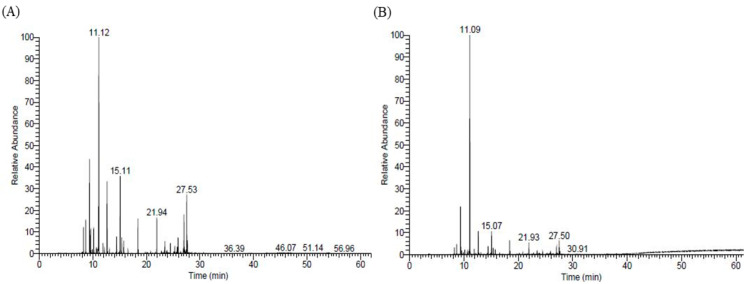
Representative GC-MS total ion chromatogram of essential oil from *A. vulgaris* extracted through hydro-distillation (**A**) and solid-phase microextraction (SPME) of the essential oil (**B**).

In the present study, 47 compounds were detected by the essential oil injection method, which accounts for 97.66% of the total area percentage (Figure 1A). Similarly, the same 47 compounds were detected by the SPME analysis of the oil, which accounts for 97.35% of the total area percentage (Figure 1B).

**Figure 2:**
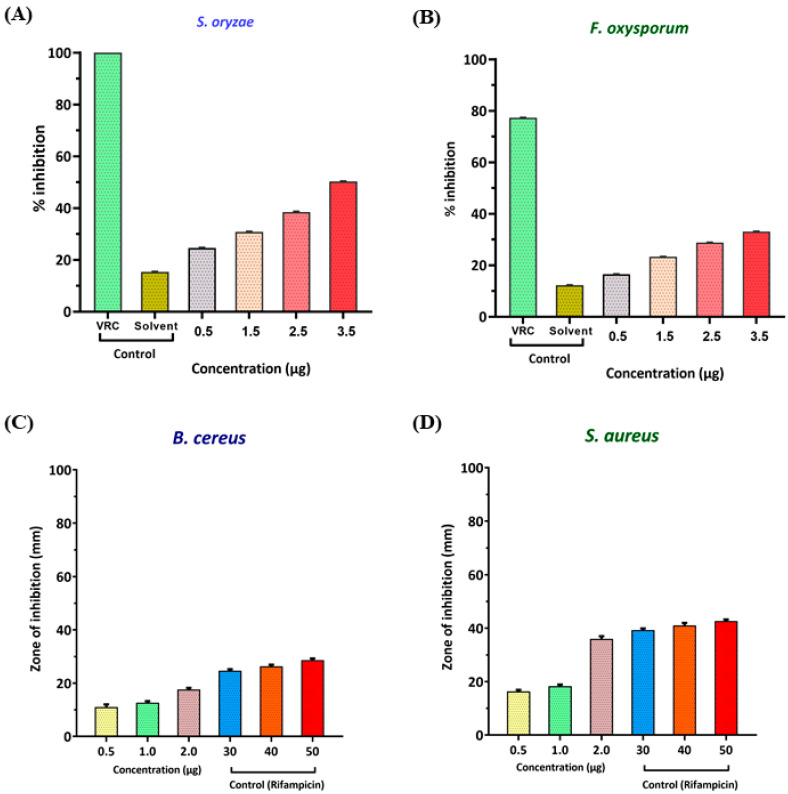
Percent inhibition of the antifungal activity of AVEO against the fungal test pathogens (**A**,**B**). Zone of inhibition of the antibacterial activity of AVEO performed on (**C**,**D**).

Using both methods, the major compounds present were eucalyptol, followed by sabinene, endo-Borneol, 2, 7-Dimethyl-2,6-octadien-4-ol, and 10-epi-ç-Eudesmol. Antifungal activities of the essential oils were evaluated against the test cultures *S. oryzae* and *F. oxysporum,* described in the Methods Section. The percent inhibition of *S. oryzae* was found to be 24.6 ± 0.09, 30.8 ± 0.09, 38.50 ± 0.09, and 50.30 ± 0.03 at 0.5 µL, 1.5 µL, 2.5 µL, and 3.5 µL of the essential oil, respectively (Figure 2A and Table 2). Furthermore, the percent inhibition of *F. oxysporum* was found to be 16.56 ± 0.06, 23.31 ± 0.06, 28.83 ± 0.06, and 33.13 ± 0.06 at 0.5 µL, 1.5 µL, 2.5 µL, and 3.5 µL of the essential oil, respectively (Figure 2B and Table 2).

**Figure 3:**
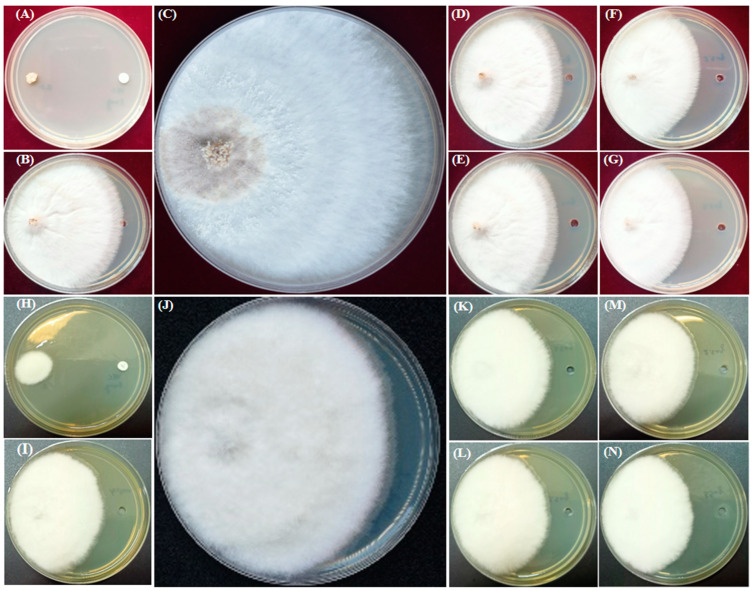
Antifungal activity of *A. vulgaris* essential oil performed on *S. oryzae* and *F. oxysporum*. Percent inhibition of the AVEO at different concentrations against *S. oryzae* on the PDA plate. Voriocanazole 1 µg as the positive control (**A**), acetone as the negative control (**B**), control plate (**C**), 0.5 µL (**D**), 1.5 µL (**E**), 2.5 µL (**F**), and 3.5 µL (**G**) of AVEO. Percent inhibition of the AVEO at different concentrations against *F. oxysporum* on the PDA plate. Voriocanazole 1 µg as the positive control (**H**), acetone as the negative control (**I**), control plate (**J**), 0.5 µL (**K**), 1.5 µL (**L**), 2.5 µL (**M**), and 3.5 µL (**N**) of AVEO.

For the positive control, voriocanazole at 1 µg exhibited a percent inhibition of 100 ± 0.0 and 77.80 ± 0.07 in the test cultures *S. oryzae* and *F. oxysporum,* respectively (Figure 3A,H). In the case of the solvent control, a percent inhibition of 15.4 ± 0.03 and 13.00 ± 0.10 in the test cultures *S. oryzae* and *F. oxysporum* was observed, respectively (Figure 3B,I). Results revealed that the essential oil showed higher antifungal activity in *S. oryzae* (Figure 3D–G) than *F. oxysporum* (Figure 3K–N) in a dose-dependent manner. The essential oil of *A. vulgaris* at 3.5 µg and the standard voriconazole at 1 µg showed 50.30% inhibition, equivalent to the efficacy of the antifungal activity against the *S. oryzae* (Figure 3G). In a similar way, the essential oil of *A. vulgaris* at 3.5 µg and the standard voriocanazole at 1 µg showed 42.58% inhibition, equivalent to the efficacy of the antifungal activity against the *F. oxysporum* (Figure 3N).

The AVEO also showed antibacterial activities in the test pathogens *B. cereus* and *S. aureus* in a dose-dependent manner. Results revealed that for *Bacillus cereus,* the zone of inhibition (mm) was 11.00 ± 1.00, 12.67 ± 0.58, and 17.67 ± 0.58 at concentrations of 0.5 µL, 1 µL, and 2 µL, respectively (Figure 2C and Table 3). The zone of inhibition of *Staphylococcus aureus* was found to be 16.33 ± 0.58, 18.33 ± 0.58, and 36.00 ± 1.00 at concentrations of 0.5 µL, 1 µL, and 2 µL, respectively (Figure 2D and Table 3).

**Figure 4:**
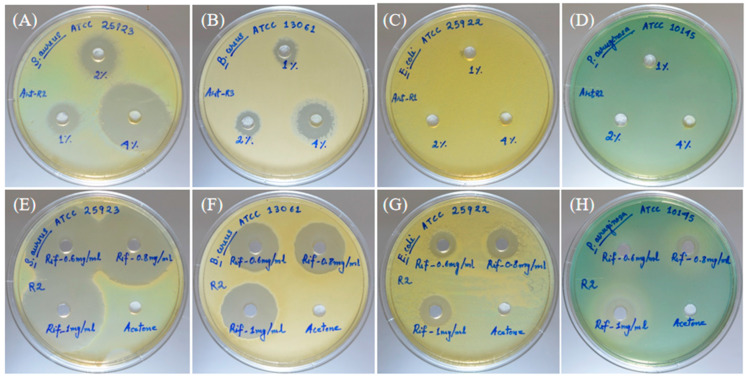
Antibacterial activity of AVEO performed on *S. aureus* and *B. cereus*. Zone of inhibition of the AVEO at different concentrations against *S. aureus* on the MHA plate: 0.5 µL, 1 µL, and 2 µL (**A**). Zone of inhibition of the AVEO at different concentrations against *B. cereus* on the MHA plate: 0.5 µL, 1 µL, and 2 µL (**B**). Antibacterial activity of essential oil performed on *E. coli* (**C**) and *P. aeruginosa* (**D**). Rifampicin as the positive control at 30, 40, and 50 µg in *E. coli* (**G**), *P. aeruginosa* (**H**), *S. aureus* (**E**), and *B. cereus* (**F**).

In the case of essential oil at 0.5, 1, and 2 µL, the zone of inhibition for *S. aureus* was 16.33 ± 0.58, 18.33 ± 1.00, and 36.00 ± 1.00 (Figure 4A and Table 3), and for *B. cereus,* the zone of inhibition was 11.00 ± 1.00, 12.67 ± 0.58, and 17.67 ± 0.58, respectively (Figure 4B and Table 1). The AVEO did not show the zone of inhibition in the test pathogens *E. coli* and *P. aeruginosa* (Figure 4C,D).

In the case of the positive control (rifampicin), at 30 µg, the zone of inhibition for *S. aureus, B. cereus, E. coli,* and *P. aeruginosa* was 39.33 ± 0.58, 24.67 ± 0.58, 14.33 ± 0.58, and 8.00 ± 0.00, respectively (Figure 4E–H and Table 3). At 40 µg, the zone of inhibition for *S. aureus, B. cereus, E. coli,* and *P. aeruginosa* was 41.00 ± 1.00, 26.33 ± 0.58, 16.00 ± 0.00, and 11.67 ± 0.58, respectively (Figure 4E–H and Table 3). Similarly, at 50 µg for *S. aureus, B. cereus, E. coli,* and *P. aeruginosa,* the zone of inhibition was 43.00 ± 0.00, 28.67 ± 0.58, 17.00 ± 0.00, and 15.00 ± 0.00, respectively (Figure 4E–H and Table 3). The solvent control did not show any zone of inhibition for the test bacterial pathogens (Figure 4E–H and Table 3).

The minimum inhibitory concentration (MIC) and minimum bactericidal concentration (MBC) of the essential oil against *B. cereus* were 0.31% and 0.63%, respectively, and for *S. aureus* the MIC and MBC were 0.63% and 2.5%, respectively. The MIC and MBC of rifampicin against *B. cereus* were ≤0.49 µg/mL and 1.95 µg/mL, respectively, and for *S. aureus,* the MIC and MBC were ≤0.49 µg/mL. In our results, the minimum inhibitory concentration for *B. cereus* was ≤0.49 µg/mL and the minimum bactericidal concentration was ≤1.95 µg/mL. In addition, for *S. aureus*, the minimum inhibitory concentration was ≤0.49 µg/mL and the minimum bactericidal concentration was ≤0.49 µg/mL (Table 4).

## 3. Discussion

The abundance of eucalyptol in the essential oils is consistent with previous findings on the *A. vulgaris* essential oil composition from Qinghai–Tibet Plateau regions [15]. Similar to this, eucalyptol was the major compound obtained in the oil isolated from the Indo Gangetic regions of India. The percentage composition of AVEO by direct injection was 6.27% and by the SPME method was 26.34% [16]. However, in the case of Egypt, camphor (13.83%) was reported as the abundant compound [17]. Analysis of essential oil from the aerial parts of *A. vulgaris* from Nepal revealed sabinene (11.29%) as the most prominent compound [18]. Differences in the prominent compound between the authors could be clarified by variables such as rainfall and season collection, plant ontogeny, as well as geographic location, plant parts, and extraction techniques [19].

*A. vulgaris* has been shown to have antimicrobial properties [20,21]. The essential oil of *A. vulgaris* had previously demonstrated significant fungicidal activity, inhibiting the mycelia growth of fungi. The zone of inhibition of the essential oils extracted from the *A. vulgaris* growing in Gorkha (862 m altitude) and Chitwan (208 m altitude) was 12 and 15 mm and 12 and 11 mm, respectively, and demonstrated effective antibacterial properties against *Klebsiella pneumonia* and *Acinetobacter baumannii* [22]. Eucalyptol is mostly employed in the prevention of chronic obstructive pulmonary disease and has antifungal activity against the candida spectrum, as the chemical compositions of *A. vulgaris* primarily have antifungal activities [23]. *Candida albican* hyphal cells are inhibited by both camphor and eucalyptol at concentrations of 0.125 mg/mL and 23 mg/mL, respectively [24]. Additionally, sabinene is a promising antifungal compound with an anti-inflammatory effect [25]. Endoborneol is used in the treatment of anxiety, fatigue, and insomnia [26]. Caryophyllene also has antifungal properties, and caryophyllene oxide works as a broad-spectrum antifungal in plants and inhibits *Fusarium moniliforme* [27,28]. Eudesmol demonstrates a significant antifungal effect at 100 ppm [29]. According to our findings, majority of the prominent compounds present in the essential oil exhibited antifungal properties.

According to our findings, the AVEO exhibited more antifungal action against *S. oryzae* than *F. oxysporum* in a dose-dependent manner. The main components present, which have the antifungal qualities mentioned earlier, could be responsible for the antifungal activity. In other *Artemisia* species such as *A. sieberi*, the essential oil showed fungistitic activities against *Fusarium moniliforme* and *Fusarium solani* [30]. *Artemisia herba alba* essential oil inhibited the mycelial growth of *Fusarium sporotrichioides* and there was a significant reduction in mycelium growth at 0.025% and 0.05% [31].

Toxicants produced by *Fusarium* spp. pose significant threats to both human health and food safety [32]. One of the most dangerous rotting agents is *F. oxysporum*, a phytopathogenic soil-borne ascomycete fungus that destroy plants by causing *Fusarium* wilt, a fatal vascular disease, and restricts plant growth and crop yield [33,34]. *S. oryzae* causes the stem rot of rice and accounts for 35% of crop losses, posing a serious danger to India’s rice production [35]. The disease can also cause considerable grain output losses of up to 80%.

In our results, the AVEO had antifungal activity in a dose-dependent manner, higher in *S. oryzae* than *F. oxysporum*. Antifungal activity can be attributed to the major compounds present, which possess the antifungal properties as described earlier. The essential oil of *Artemisia sieberi* was effective against *Fusarium moniliforme* and *Fusarium solani*. The oil of *A. sieberi* showed fungistatic activity against *Fusarium moniliforme* and *Fusarium solani* [30]. With different concentrations of essential oil extracted from *Artemisia herba-alba*, antifungal activity was revealed via the reduction of mycelial growth in *Fusarium sporotrichioides*. Significance reductions in mycelium growth have been observed at 0.025% and 0.05% [31].

Pathogenic bacteria such as *E. coli, P. aeruginosa, B. cereus*, and *S. aureus* cause food-borne illness and ongoing challenges in public health [36]. *S. aureus* causes food-borne disease and *B. cereus* is a common bacterium that causes gastrointestinal illness [37]. An Iranian *A. scoparia* extract was found to have an inhibitory zone (13.6 mm) against *S. aureus* but not *P. aeruginosa* [20]. Methanolic extracts of *A. vulgaris* have antibacterial activities and showed strong MIC values [38]. As in our study, the essential oil of *A. vulgaris* possesses antibacterial activity and is effective against *S. aureus* and *B. cereus* but is not effective against *E. coli* and *P. aeruginosa*.

## 4. Materials and Methods

### 4.1. Plant Material

The fresh leaves of *A. vulgaris* were collected in June 2022 from the hillocks of Phayeng Region located in the Imphal west district of Manipur (N 24° 16.275, E 093° 52.651, at an elevation of 874 m above sea level) for the analysis of essential oil chemical compositions and their antimicrobial effect.

### 4.2. Hydro-Distillation Apparatus and Methods

A Clevenger apparatus was used to hydro-distill the fresh leaves (500 g) for 4 h. The essential oils were isolated, dried with anhydrous sodium sulphate, and kept in a freezer until needed. The weight of the oil obtained per 500 g of fresh leaves was used to measure essential oil yield. The AVEO yield was 0.75% (*w/v*).

### 4.3. Headspace SPME Extraction

The AVEO collected were packed in a 10 mL clear vial which has a screw-top hole cap with silicone septa. The SPME fiber (75 µm CAR/PDMS, fused Silica 23 Ga, black plain) was then inserted and exposed to the AVEO (2 mL) contained in the clear glass for 10 min by using an SPME holder (57330-U). The fiber was then introduced into the GC for analyzing the volatile organic compounds.

### 4.4. GC and GC-MS Analysis

The volatile organic compounds from the essential oil were analyzed by using two different methods, direct injection of the AVEO and SPME. For direct injection, 0.5 µL (1:100, AVEO: n-hexane) of the AVEO was used, and for SPME, the fiber was injected for 2 min in the GC-MS. Gas chromatography–mass spectrometry analysis was employed using Trace 1300 (GC) interfaced with a TSQ DUO (MS) fitted with a TG-5MS fused silica capillary column (30 m × 0.25 mm; 0.25 μm film thickness) under an optimized condition. For GC, the oven temperature range was programmed from 40° to 280 °C, at 5 °C min^−1^, and helium was used as a carrier gas at a flow rate of 1.0 mL min^−1^ for the analysis. For the mass detector, the mass transfer line and the ion source temperature were set at 250 °C and 280 °C, respectively. The inlet injector temperature was set at 240 °C with a split mode of 1:20 maintained. The mass spectra were taken at 70 eV with a mass range filtered from 35 to 450 Mw [39].

### 4.5. Test Organisms

The antifungal activity of the AVEO was evaluated by using two fungal cultures, namely *Sclerotium oryzae* (ITCC 4107) and *Fusarium oxysporum* (MTCC 9913). For antibacterial activity, four bacterial cultures were used, namely *Bacillus cereus* (ATCC 13061), *Staphylococcus aureus* (ATCC 25923), *Escherichia coli* (ATCC 25922), and *Pseudomonas aeruginosa* (ATCC 10145).

### 4.6. Antifungal Activity of Essential Oil

The antifungal activity was performed in Potato Dextrose Agar (PDA) medium in the petri dish, with a diameter of 90 mm. One well, 5 mm in diameter, was bored 10 mm away from the periphery of the petri dish with a sterile metal cork-borer on the PDA plate, and on the opposite side a 5 mm-diameter, seven-day grown fungal pathogen was placed 10 mm away from the edge of the petri dish. Sample volumes of 50 µL of the essential oil, 1%, 3%, 5%, and 7% dissolved in acetone (*v/v*), was added in the wells, which yielded the final concentrations of 0.5, 1.5, 2.5, and 3.5 µL of essential oil in the wells, respectively. For control plates, vorioconazole (1 µg disc) was used as a positive control and acetone was used as the solvent (negative) control. The plates were kept in the refrigerator for an hour to left to diffuse the essential oils and the antifungal agent, and then the plates were incubated at 28 ± 1 °C for 5–7 days. The experiments were conducted in triplicates. After incubation, the radial mycelial growth was measured and percent inhibition was calculated using the formula below. Results are shown in mean ± SD.

% inhibition = C−T /C × 100, where C = radial growth of the pathogen in the control, and T = radial growth of the pathogen in the presence of the essential oil test sample.

### 4.7. Antibacterial Activity of the Essential Oil

The antibacterial test was performed using the agar well-diffusion method. The bacterial inoculums were prepared with turbidity equivalent to 0.5 McFarland standard (1.5 × 10^8^ CFU/mL) in sterile normal saline. The prepared inoculums of different bacteria were uniformly spread in separate Mueller–Hinton Agar (MHA) medium with the help of sterile cotton swabs, and the processes were repeated thrice, rotating the plate at an angle of 60° between each streaking. The wells were bored in the MHA plates, 90 mm in diameter, with a sterile metal cork-borer having a diameter of 6 mm. Then, 50 µL of 1%, 2%, and 4% of essential oil dissolved in acetone (*v/v*) were loaded to the wells, which yielded the final concentrations of 0.5, 1.0, and 2.0 µL of essential oil in the wells, respectively. Rifampicin (0.6 mg/mL, 0.8 mg/mL, and 1 mg/mL) was used as the positive control and acetone was used as the solvent or negative control. The essential oil and antibiotic used were allowed to diffuse for an hour in the refrigerator and then plates were incubated at 37 °C for 24 h. All the experiments were performed in triplicates. The zones of inhibition were then measured with the help of a scale. Results are shown in mean ± SD.

### 4.8. Minimum Inhibitory Concentration (MIC) and Minimum Bactericidal Concentration (MBC) of the Essential Oil

The Gram-positive test organisms *B. cereus* and *S. aureus* which yielded an inhibition zone were chosen to assay MIC via the broth microdilution method using 96-well microplates. Selected test holes for the assay in the microplate were filled with 100 µL of Mueller–Hinton Broth (MHB) and performed two-fold serial dilution of 20% essential oil of *Artemisia vulgaris* dissolved in acetone (*v/v*) to obtain final concentrations of 10%, 5%, 2.5%, 1.25%, 0.63%, 0.31%, 0.16%, 0.08%, 0.04%, and 0.02%. For the antibiotic (rifampicin), a 500 µg/mL concentration was used for serial dilution to obtain a final concentration of 250, 125, 62.50, 31.25, 15.63, 7.81, 3.91, 1.95, 0.98, and 0.49 µg/mL. A volume of 10 µL of the inoculum of the test organism with turbidity equivalent to 0.5 McFarland standards was added to the test holes [40]. Broth with inoculum was used for growth control and broth without inoculums was used for negative growth control. The microplates were incubated at 37 °C for 24 h. For determining the MBC, after incubation, 10 µL of the culture from different concentrations was streaked in Mueller–Hinton Agar (MHA) plates and incubated at 37 °C for 24 h. All the experiments were performed in triplicate. The lowest concentration of the essential oil inhibiting the visible growth of the test organism was taken as the MIC and the lowest concentration of the essential oil which did not show any growth in the agar plate was taken as the MBC.

## 5. Conclusions

The identification of the essential oil via GC/MS analysis revealed eucalyptol as the most dominant compound present in the leaves of *A. vulgaris*. This study has confirmed that there are variations in the VOC’s profile when compared with that of the same plant species grown in different ecological sites. In addition, differences observed in the diversity of essential oil produced may be due to seasonal variation, rainfall patterns, and geographical locations. Results revealed that the essential oil characterized by the hydro-distillation and SPME extraction yielded the same chemical profile. The essential oil of *Artemisia vulgaris* possesses antimicrobial activities. The essential oils of *A. vulgaris*, when compared with standard voriocanazole, showed 50.3% and 43.02% equivalent efficacy of the antifungal activity against the *S. oryzae* and *F. oxysporum,* respectively. The essential oil was more effective against *S. aureus* than *B. cereus* in antibacterial activity. The antimicrobial activities of *A. vulgaris* can be further investigated to utilize as a source of natural antimicrobial drugs.

## Figures and Tables

**Table 1:** Chemical compositions of the AVEO extracted by the SPME method and the direct AVEO injection method.

	Direct AVEO Injection Method					AVEO SPME Method				
S No.	^a^ RT	Compound	^b^ RSI	^C^ RA%	Mol. Wt.	^a^ RT	Compound	^b^ RSI	^C^ RA%	Mol. Wt.
1	8.23	α-Pinene	948	2.24	136	8.23	α-Pinene	949	1.45	136
2	8.65	Camphene	967	3.03	136	8.65	Camphene	964	2.14	136
3	9.38	Sabinene	950	8.44	136	9.37	Sabinene	938	8.86	136
4	9.47	p-mentha-1(7)	923	1.27	136	9.46	p-mentha-1(7)	927	1.34	136
5	9.57	1-Octen-3-ol	920	2.1	128	9.56	1-Octen-3-ol	894	0.78	128
6	9.88	β-Myrcene	867	0.45	136	9.88	β-Myrcene	867	0.41	136
7	10.16	Yomogi alcohol	916	2.34	154	10.15	Yomogi alcohol	911	0.93	154
8	10.63	1,3-Cyclohexadiene,1-methyl-4-(1-methylethyl)-	934	0.53	136	10.62	1,3-Cyclohexadiene,1-methyl-4-(1-methylethyl)-	931	0.63	136
9	10.87	o-Cymene	967	0.43	134	10.87	o-Cymene	967	0.52	134
10	11.00	Cyclohexene,1-methyl-5-(1-methylethenyl)-,(R)-	910	0.81	136	10.99	Cyclohexene,1-methyl-5-(1-methylethenyl)-,(R)-	907	1.06	136
11	11.12	Eucalyptol	923	29.91	154	11.08	Eucalyptol	937	47.30	154
12	11.88	γ-Terpinene	923	0.85	136	11.88	γ-Terpinene	923	1.12	136
13	12.15	5-Isopropyl-2-methylbicyclo [3.1.0]hexan-2-ol	925	0.45	154	12.15	5-Isopropyl-2-methylbicyclo [3.1.0]hexan-2-ol	921	0.29	154
14	12.65	2,7-Dimethyl-2,6-octadien-4-ol	916	6.76	154	12.63	2,7-Dimethyl-2,6-octadien-4-ol	918	4.24	154
15	12.76	Cyclohexene,1-methyl-4-(1-methylethylidene)-	955	0.15	136	12.76	Cyclohexene,1-methyl-4-(1-methylethylidene)-	952	0.27	136
16	13.09	Linalool	892	0.4	154	13.09	Linalool	875	0.28	154
17	14.29	1,7-Octadien-3-one,2-methyl-6-methylene-	858	0.1	150	14.29	1,7-Octadien-3-one,2-methyl-6-methylene-	860	0.16	150
18	14.43	(-)-Alcanfor	965	1.37	152	14.43	(-)-Alcanfor	966	1.78	152
19	14.81	Isobornyl formate	865	0.12	154	14.81	Isobornyl formate	884	0.18	182
20	15.00	trans-Verbenol	887	0.22	152	14.97	trans-Verbenol	844	0.28	152
21	15.11	endo-Borneol	939	8.24	154	15.07	endo-Borneol	946	4.76	154
22	15.39	Terpinen-4-ol	896	1.21	154	15.38	Terpinen-4-ol	906	1.32	154
23	15.77	α-Terpineol	942	0.91	154	15.76	α-Terpineol	951	0.83	154
24	16.57	trans-Carveol	886	0.26	152	16.56	trans-Carveol	889	0.34	152
25	18.41	Bornyl acetate	955	2.92	196	18.40	Bornyl acetate	950	2.74	196
26	19.76	Cyclohexene,1,5,5-trimethyl-3-methylene-	903	0.11	136	19.76	Cyclohexene,1,5,5-trimethyl-3-methylene-	900	0.16	136
27	20.14	Tricyclo [5.4.0.0(2,8)]undec-9-ene, 2,6,6,9-tetramethyl-,(1R,2S,7R,8R)-	866	0.11	204	20.14	Tricyclo [5.4.0.0(2,8)]undec-9-ene, 2,6,6,9-tetramethyl-,(1R,2S,7R,8R)-	871	0.28	204
28	20.78	Copaene	871	0.21	204	20.79	Copaene	885	0.36	204
29	21.15	cis-β-Copaene	912	0.10	204	21.15	cis-β-Copaene	900	0.17	204
30	21.69	1H-Cycloprop[e]azulene,1a,2,3,4,4a,5,6,7b-octahydro-1,1,4,7-tetramethyl-,	904	0.13	204	21.68	1H-Cycloprop[e]azulene,1a,2,3,4,4a,5,6,7b-octahydro-1,1,4,7-tetramethyl-,	909	0.24	204
31	21.94	Caryophyllene	204	2.95	945	21.93	Caryophyllene	945	2.48	204
32	22.79	Humulene	204	0.26	204	22.78	Humulene	900	0.27	204
33	23.33	1-Methyl-4-(6-methylhept-5-en-2-yl)cyclohexa-1,3-diene	892	0.13	204	23.33	1-Methyl-4-(6-methylhept-5-en-2-yl)cyclohexa-1,3-diene	885	0.11	204
34	23.46	Germacrene D	952	1.01	204	23.46	Germacrene D	951	0.74	204
35	23.74	cis-Muurola-4(15),5-diene	944	0.15	204	23.74	cis-Muurola-4(15),5-diene	925	0.18	204
36	23.85	Bicyclogermacrene	906	0.43	204	23.84	Bicyclogermacrene	912	0.49	204
37	24.45	1-Isopropyl-4,7-dimethyl-1,2,3,5,6,8a-hexahydronaphthalene	927	0.84	204	24.44	1-Isopropyl-4,7-dimethyl-1,2,3,5,6,8a-hexahydronaphthalene	923	0.53	204
38	25.31	1,6,10-Dodecatrien-3-ol,3,7,11-trimethyl-, (E)-	923	0.65	222	25.31	1,6,10-Dodecatrien-3-ol,3,7,11-trimethyl-	914	0.28	222
39	25.75	5,10-Pentadecadiyn-1-ol	953	0.77	220	25.75	5,10-Pentadecadiyn-1-ol	951	0.33	220
40	25.93	Caryophyllene oxide	920	1.38	220	25.92	Caryophyllene oxide	912	0.61	220
41	26.39	(-)-Globulol	902	0.18	222	26.39	(-)-Globulol	895	0.11	222
42	26.89	2-Methyl-3-(3-methyl-but-2-enyl)-2-(4-methyl-pent-3-enyl)-oxetane	930	0.35	222	26.87	2-Methyl-3-(3-methyl-but-2-enyl)-2-(4-methyl-pent-3-enyl)-oxetane	937	0.15	222
43	27.02	Cedren-13-ol, 8-	870	4.12	220	27.01	Cedren-13-ol, 8-	875	1.77	220
44	27.14	11,11-Dimethyl-4,8-dimethylenebicyclo [7.2.0]undecan-3-ol	220	0.36	220	27.13	11,11-Dimethyl-4,8-dimethylenebicyclo [7.2.0]undecan-3-ol	901	0.22	220
45	27.24	Dihydro-cis-à-copaene-8-ol	943	0.28	222	27.23	Dihydro-cis-à-copaene-8-ol	945	0.19	222
46	27.53	10-epi-γ-Eudesmol	949	6.50	222	27.5	10-epi-γ-Eudesmol	940	3.09	222
47	27.67	10-Epijuneol	920	1.13	264	27.65	10-Epijuneol	919	0.58	264
		**Total area % = 97.66**					**Total area % = 97.35**			

^a^ Retention time. ^b^ Reverse search index on TG-5MS capillary column. ^c^ Relative area (peak area relative to the total peak area).

**Table 2:** Antifungal activity of *A. vulgaris* essential oils.

S No.	Sample	Conc.	Amount of E.O. per 50 µL Volume	Percent Inhibition (Mean ± SD)	
				S. oryzae ITCC 4107	F. oxysporum MTCC 9913
1.	Essential oil (E.O.)	1%	0.5 µL	24.6 ± 0.09	16.56 ± 0.06
		3%	1.5 µL	30.8 ± 0.09	23.31 ± 0.06
		5%	2.5 µL	38.50 ± 0.09	28.83 ± 0.06
		7%	3.5 µL	50.3 ± 0.03	33.13 ± 0.06
2.	Vorioconazole	1 µg	-	100 ± 0.0	77.8 ± 0.07
3.	Acetone	-	-	15.4 ± 0.03	13.0 ± 0.1

-: Not applicable.

**Table 3:** Antibacterial activity of *A. vulgaris* essential oil.

S No.	Sample	Conc.	Amount of AVEO/Antibiotic per 50 µL Volume	Zone of Inhibition (mm), Mean ± SD			
				*S. aureus* ATCC 25923	*B. cereus* ATCC 13061	*E. coli* ATCC 25922	*P. aeruginosa* ATCC 10145
1.	Essential oil (E.O.)	1%	0.5 µL	16.33 ± 0.58	11.00 ± 1.00	Nil	Nil
		2%	1 µL	18.33 ± 0.58	12.67 ± 0.58	Nil	Nil
		4%	2 µL	36.00 ± 1.00	17.67 ± 0.58	Nil	Nil
2.	Rifampicin	0.6 mg/ml	30 µg	39.33 ± 0.58	24.67 ± 0.58	14.33 ± 0.58	8.00 ± 0.00
		0.8 mg/ml	40 µg	41.00 ± 1.00	26.33 ± 0.58	16.00 ± 0.00	11.67 ± 0.58
		1.0 mg/ml	50 µg	43.00 ± 0.00	28.67 ± 0.58	17.00 ± 0.00	15.00± 0.00
3.	Acetone	-	-	Nil	Nil	Nil	Nil

-: Not applicable.

**Table 4:** MIC and MBC of the essential oil of *A. vulgaris*.

S No.	Sample	Microorganisms	MIC *	MBC **
1.	Essential oil	*B. cereus* *S. aureus*	0.31% 0.63%	0.63% 2.5%
2.	Rifampicin	*B. cereus* *S. aureus*	≤0.49 µg/mL ≤0.49 µg/mL	1.95 µg/mL ≤0.49 µg/mL

* Minimum inhibitory concentration. ** Minimum bactericidal concentration.
